# Letter to the Editor Re: A population study of screening history and diagnostic outcomes of women with invasive cervical cancer

**DOI:** 10.1002/cam4.4240

**Published:** 2021-08-26

**Authors:** Philip E. Castle

**Affiliations:** ^1^ Division of Cancer Prevention National Cancer Institute National Institutes of Health Rockville Maryland USA; ^2^ Division of Cancer Epidemiology and Genetics National Cancer Institute National Institutes of Health Rockville Maryland USA

Benard et al.^1^ reported on the screening and follow‐up care of women prior to being diagnosed with cervical cancer, using data from three population‐based cancer registries. They found 60% of women diagnosed with cervical cancers had not been screened in the 6 months to 5 years prior to diagnosis, confirming previous reports more than 50% of cervical cancers are diagnosed in under‐/un‐screened women.^2^, ^3^, ^4^ Another one‐third of the cases with adequate screening did not have adequate follow‐up of their positive screen. Taken together, and assuming screening with proper follow‐up works as well (albeit imperfectly) in all women, many additional cervical cancers could be averted by increasing access and adherence to recommended screening and follow‐up management of screen positives. Lack of screening and losses to follow‐up for care of screen‐positive women were strongly associated with lower income and being uninsured, indicators of lower socioeconomic status, a key underpinning of health disparities.

It was notable that not only did a high percentage of those cancers occur in women who did not get screened in the last 5 years, those cases tended to be diagnosed at a more advanced stage (*p*_trend_ =0.002). A possible implication of the more advanced stage cancers in the under‐/un‐screened population is a greater mortality due to those cancers. However, the degree of difference in the stage distribution is confounded by the enrichment of cervical adenocarcinoma (ADCA) in the screened population, which as noted by Benard et al.^1^ tended to be diagnosed at a later stage than squamous cell carcinoma (SCC). The stage difference for SCC, the histology for which screening is effective, and therefore the health inequities between screened and under‐/un‐screened population was likely greater still.

These data also highlight one of the opportunities, in addition to increasing screening in underserved, underscreened populations, for improving cervical cancer prevention, which is the prevention and early detection of ADCA. As noted by Benard et al.^1^ and previously,^3^, ^5^ well‐screened populations have a higher ADCA:SCC than poorly screened populations.

Past and current screening algorithms rely on cytology as the primary screening test or as a triage test, which is ineffective in the prevention and even the early detection of cervical adenocarcinoma. In fact, the highest ADCA:SCC tends to be found in those populations with negative screening history^1^, ^3^; in this study,^1^ 26 cases of cancer were diagnosed in insured women with all normal screens, most of whom likely were screened by cytology alone (only 38.5% had had any HPV test). Of these cases, the ADCA:SCC was 1:1 and only 46% of these cancers were SCC. By comparison, 57% of cases diagnosed in those with positive screen and adequate follow‐up were SCC and 78% of cases diagnosed in the under‐/un‐screened were SCC.

Thus, screening strategies must be improved, especially for precursors of ADCA like adenocarcinoma in situ (AIS), if we are to make further advances in cervical cancer prevention. Although biomarkers such detection of HPV16 and HPV18, which are included in current management guidelines,^6^ will help identify women at risk for ADCA, the PPV will be too low for immediate treatment and thus colposcopy will be needed. However, identification of precursors such as AIS remains a clinical challenge because their location in the endocervical canal precludes their visualization and the thin tissue there often results in inadequate sampling for diagnostic evaluation.

Although prophylactic HPV vaccines will likely address this issue in the long term, there are several generations of at‐risk, persistently HPV‐infected women for whom HPV vaccination will have little or no benefit, some of whom will get cervical cancer and notably ADCA despite the availability of effective cervical cancer screening. Thus, in addition to increasing access and adherence to screening and follow‐up care to reduce cancer health inequities, more effective screening algorithms are needed if we hope to achieve further reductions in this highly preventable cancer.

## DISCLAIMER

The opinions expressed by the authors are their own and this material should not be interpreted as representing the official viewpoint of the US Department of Health and Human Services, the National Institutes of Health or the National Cancer Institute.

## Data Availability

Data sharing is not applicable to this article as no new data were created.
